# Preferential Mapping of Sex-Biased Differentially-Expressed Genes of Larvae to the Sex-Determining Region of Flathead Grey Mullet (*Mugil cephalus*)

**DOI:** 10.3389/fgene.2020.00839

**Published:** 2020-08-21

**Authors:** Lior Dor, Andrey Shirak, Arie Y. Curzon, Hana Rosenfeld, Iris M. Ashkenazi, Oriya Nixon, Eyal Seroussi, Joel I. Weller, Micha Ron

**Affiliations:** ^1^Institute of Animal Science, Agricultural Research Organization, Bet Dagan, Israel; ^2^Robert H. Smith Faculty of Agriculture, Food and Environment, The Hebrew University of Jerusalem, Rehovot, Israel; ^3^National Center for Mariculture, Israel Oceanographic and Limnological Research, Eilat, Israel

**Keywords:** *Mugil cephalus*, sex determining region, comparative mapping, gene enrichment analysis, heterogametic males, expression, candidate genes

## Abstract

Flathead gray mullet (*Mugil cephalus*) is a cosmopolitan mugilid species popular in fishery and aquaculture with an economic preference for all-female population. However, it displays neither sexual dimorphisms nor heteromorphic sex chromosomes. We have previously presented a microsatellite-based linkage map for this species locating a single sex determination region (SDR) on linkage group 9 (LG9) with evidence for XX/XY sex determination (SD) mechanism. In this work, we refine the critical SDR on LG9, and propose positional- and functional- candidate genes for SD. To elucidate the genetic mechanism of SD, we assembled and compared male and female genomic sequences of 19 syntenic genes within the putative SDR on mullet’s LG9, based on orthology to tilapia’s LG8 (tLG8) physical map. A total of 25 sequence-based markers in 12 genes were developed. For all markers, we observed association with sex in at least one of the two analyzed *M. cephalus* full-sib families, but not in the wild-type population. Recombination events were inferred within families thus setting the SDR boundaries to a region orthologous to ∼0.9 Mbp with 27 genes on tLG8. As the sexual phenotype is evident only in adults, larvae were assigned into two putative sex-groups according to their paternal haplotypes, following a model of XY/XX SD-system. A total of 107 sex-biased differentially expressed genes in larvae were observed, of which 51 were mapped to tLG8 (48% enrichment), as compared to 5% in random control. Furthermore, 23 of the 107 genes displayed sex-specific expression; and 22 of these genes were positioned to tLG8, indicating 96% enrichment. Of the 27 SDR genes, *BCCIP*, *DHX32A*, *DOCK1*, and *FSHR* (*GTH-RI*) are suggested as positional and functional gene candidates for SD.

## Introduction

Teleostei is a large infraclass including about 34,200 fish species^1^. It presents diversity of mechanisms for sex determination (SD), which involve environmental and social effects, genetic and epigenetic influences (Devlin and Nagahama, 2002; Baroiller et al., 2009; Martinez et al., 2014; Mei and Gui, 2015). In this clade, heterogametic male species (XY) are found along with heterogametic female species (WZ). For example, XX/XY SD-system has been detected in ***Poecilia reticulata*** (Winge, 1922), ***Oryzias latipes*** (Matsuda et al., 2002) and ***Oreochromis niloticus*** (Eshel et al., 2014), while WZ/ZZ SD-system has been found in ***Cynoglossus semilaevis*** (Zhuang et al., 2006), ***Oreochromis aureus*** (Don and Avtalion, 1988) and ***Parodon hilarii*** (Moreira-Filho et al., 1993). However, among different animal species only several genes have been established as initiators of the SD cascade that finally results in the two alternative forms of female and male. Such master genes of SD frequently have truncated copies of the original genes i.e., ***AMH***, ***DMRT1***, ***SOX3***, ***IRF9***, ***AMHR2*,** and ***GSDF*** (Li and Gui, 2018; Matsuda, 2018). The truncated copies may be located in tandem, adjacent to the original genes, like the ***AMHY*** and ***AMH*** in Nile tilapia (Eshel et al., 2014; Li et al., 2015; Curzon et al., in press), or in different chromosomes, like ***DMRTY*** and ***DMRT1*** in medaka (Matsuda et al., 2002). Likewise, the mammalian master SD regulator ***SRY*** is probably a male-specific copy of ***SOX3*** (Waters et al., 2007). Moreover, ***SOX3*** is the master SD gene in ***Oryzias dancena*** (Takehana et al., 2014). The salmonid master SD regulator Sdy is probably a male-specific copy of IRF9 (Yano et al., 2012). Multiple copies of AMHR2 and GSDF play a key role in SD of Tiger Pufferfish (T***akifugu rubripes***) and ***Oryzias luzonensis***, respectively (Kamiya et al., 2012; Myosho et al., 2012). Although fine mapping of SD region (SDR) has been pursued in a number of fish species; e.g., ***Takifugu niphobles***, ***Channa argus***, ***Seriola dorsalis***, ***Poecilia reticulata*** and most of tilapia species; none of the above six master genes of SD have been detected in their SDR (Gammerdinger et al., 2016; Ieda et al., 2018; Purcell et al., 2018; Dor et al., 2019; Wang et al., 2019).

Sex-biased gene expression is known to affect development of sexually dimorphic traits. Differential analysis of male and female gene expression is an important tool for studying determination and differentiation of sex. RNA-seq and transcriptome assembly have been implemented for many fish species e.g., *Poecilia reticulata* (Sharma et al., 2014), *Oreochromis niloticus* (Tao et al., 2013), *Atractosteus tropicus* (Amores et al., 2011), and salmonid species (Carruthers et al., 2018).

Flathead gray mullet (***Mugil cephalus***) is the most widespread species among the family ***Mugilidae***, which occupies a wide variety of marine, estuarine and freshwater environments, although spawning occurs in the sea (Whitfield et al., 2012). It is a popular fishery and aquaculture species of a considerable commercial value and the demand for mullet roe in many parts of the world has grown considerably in recent decades (Hung and Shaw, 2006). Flathead gray mullet does not display sexual dimorphism. However, females are preferred due to their higher growth rate and ovaries of high economic value (Aizen et al., 2005; Katselis et al., 2005; Hung and Shaw, 2006). In our previous study, we generated the first draft of linkage map of the gray mullet and found synteny relationships between flathead gray mullet and Nile tilapia linkage groups (LGs) (Dor et al., 2016). Segregating markers on LGs 1, 3, and 23 were associated with sex in different tilapia species (Curzon et al., in press). Nevertheless, we located a single SDR on mullet LG9, orthologous to tilapia LG8 (tLG8), with evidence for XX/XY SD mechanism (Dor et al., 2016). In the current study we refine the SDR on LG9 and propose positional candidate genes for SD by expression and putative function.

## Materials and Methods

### Fish Samples

Five types of samples were collected and used in the present study: (1) DNA of two unrelated full-sib families: A1 (24 males and 24 females) and B (14 males and 12 females) (Dor et al., 2016), (2) Fin clips of wild type (WT) *M. cephalus* (50 males and 50 females) were collected from rivers estuaries along the Israeli shore of the Mediterranean Sea, (3) Four gonads (2 females and 2 males) and 3 brains (2 females and 1 male), of F_2_ generation fishes (3 years) of captivated population, originally obtained from the Israeli Mediterranean coast. The population was maintained at the National Center for Mariculture (NCM) under ambient hatchery, and provided balanced 1:1 male:female ratio in progeny (Aizen et al., 2005; Meiri-Ashkenazi et al., 2011), (4) 45 larvae (28 days post hatching, dph) were sampled from single spawning tank of NCM (Aizen et al., 2005), progeny of a single *M. cephalus* female and 3 males, and stored in “RNA later” solution for DNA and RNA extraction (Invitrogen, Carlsbad, CA, United States), (5) Fin clips of the latter parents (female and three males) were stored in ethanol for DNA extraction and parenthood analysis. Prior to all handling procedures, fish were anesthetized in 0.07% clove oil (Frutarom, Haifa, Israel).

### DNA and RNA Extraction

DNA was extracted from fin samples using the MasterPure^TM^ DNA Purification Kit (Epicenter^®^ Biotechnologies, Madison, WI, United States) following the manufacturer’s recommended protocol. Total DNA and RNA were extracted from each larva sample using TRI Reagent^®^ (Sigma-Aldrich, St. Louis, MO, United States) following the manufacturer’s protocol. Total RNA was extracted from the mature gonads and brains samples using RNeasy Mini Kit (Qiagen, Hilden, Germany) according to the manufacturer’s instructions. DNA and RNA concentrations were quantified using a NanoDrop 1000 Spectrophotometer (Thermo Scientific, United States). DNA samples with sufficient DNA quantity were diluted to 20 ng/μl. DNA samples of larvae and potential parents were distributed into 96-well PCR plates for parenthood tests. RNA integrity of larvae, brains and gonads’ samples were assessed using TapeStation 2200 (Agilent Technologies, Santa Clara, CA, United States).

### Deep Sequencing and Assembly of the *M. Cephalus* Genome

DNA samples of male and female from family A1 were used for deep sequencing. The male sample has been sequenced and assembled previously (Dor et al., 2016) and the female sample was sequenced using Illumina 260-bp paired-end technology (HiSeq2500, Illumina, Redwood City, CA, United States). The data obtained was deposited in the sequence read archive (ENA experiment accession no: ERX3552876). The genome reads were assembled using SOAPdenovo2 modules^2^ in four steps as follows: (1) the data were trimmed with TrimmomaticPE^3^ using parameters LEADING:30, TRAILING:30, MINLEN:50; (2) the frequency of Kmers in the trimmed output was calculated using the SOAPdenovo2 module KmerFreq_AR under the options -k 17 -t 8 -q 33; (3) the output of the KmerFreq_AR module was used to correct sequence errors in the data reads using the Corrector_AR module with -r 30 -t 8 switches; and (4) the sequence reads were assembled by applying the command line “SOAPdenovo-63mer all -s config_file -K 35 -m 51 -p 8.” The config file included the lines: max_rd_len = 310, avg_ins = 400, reverse_seq = 0, asm_flags = 3, rank = 1 and the access paths for the files with the trimmed and corrected sequence reads of the forward and reverse mate pairs (q1, q2).

### Sex-Determination Region Construction

Dor et al. (2016) mapped the *M. cephalus* SDR to LG9 in homology to Nile tilapia (*O. niloticus*) region encompassing 18.02 to 19.80 Mbp on tLG8. We downloaded a 1.78 Mbp tilapia sequence from NCBI database (Genome build Orenil1.1). To build all relevant scaffolds, the sequence was locally BLAST queried against the flathead gray mullet male and female assembled sequencing data. The resulting flathead gray mullet scaffolds were BLAST queried against all teleost sequences in NCBI. This yielded the highest scores for seabass (*Dicentrarchus labrax*) LG1 sequences.

### Markers Development and Sequencing

Each tested gene was separately assembled using GAP5 (Bonfield and Whitwham, 2010) applying the following procedure. (1) a reference genomic *D. labrax* sequence was used and exons were tagged; (2) all flathead gray mullet male and female scaffolds were separately queried against *D. labrax* reference gene sequence; (3) to derive an independent *M. cephalus* gene sequence, the resulting sequences hits were exported and *de novo* assembled with GAP4 (Staden et al., 2000) considering their exon content; and (4) male and female sequences were compared to detect sex-specific polymorphism. Primers were designed for each detected polymorphism using PRIMER3 (Untergasser et al., 2012). The designed primers were tested on selected samples of 8 males and 9 females of the two families. PCR reactions were performed in a total volume of 10 μl using GoTaq^®^ G2 Green Master Mix (Promega Corporation, Beit Haemek, Israel), and 5 pmol of each primer (IDT, Jerusalem, Israel). Amplification program was: initial denaturation at 94°C for 3 min, 30 cycles at 93°C for 40 s, 63°C for 40 s, 72°C for 1 min, and final extension step at 72°C for 10 min. The amplicons were separated by electrophoresis on 2% agarose gel and visualized by staining with ethidium bromide (1.5 μg/ml) by UV light. Fragments were purified from agarose gel using a DNA Gel Extraction Kit (EMD Millipore, Billerica, MA, United States) and sequenced at the life sciences core facilities of Weizmann Institute of Science, Rehovot, Israel.

### Linkage and Physical Mapping of the SDR

Microsatellite markers fhm 558 and fhm560 that are flanking the SDR (Figure 2, Dor et al., 2016) were BLAST queried against *D. labrax* genome. A 1.5 Mbp homologous region between 8.75 and 10.25 Mbp was found on LG1 harboring 62 genes. Nineteen genes were assembled based on *M. cephalus* deep sequencing data of which 12 showed polymorphism suitable for the development of SNP markers (Figure 2). A total of 24 primer pairs were tested (Supplementary Table S1). 15 polymorphic microsatellites markers, segregating in both families A1 and B, based on XY/XX sex-determination system, were selected for fine mapping of the SDR (Dor et al., 2016; Figure 2). These markers were genotyped for 9 and 8 individuals of families A1 and B, respectively. Recombinants and non-recombinants were inferred based on sex-associated paternal haplotypes.

### Parenthood Test of Larvae

All 45 larvae and their potential parents of a single female and 3 males, were genotyped using a panel of 6 previously developed microsatellite markers located in the SDR: fhm 421, 528, 552, 553, 558, and 560 (Dor et al., 2016). All markers were separately amplified by a two-step PCR following Dor et al. (2014). The fluorescently dyed PCR products of all tested markers were detected by an ABI3130 Genetic Analyzer and genotyped with GeneMapper software v.4.0 (Applied Biosystems, Foster City, CA, United States) using GeneScan-500 LIZ size standard (Applied Biosystems, Foster City, CA, United States). Potential parents not sharing an allele with progeny for all genetic markers were excluded as parents based on the “exclusion principle” (Jamieson and Taylor, 1997).

### RNA Sequencing

Twenty-four RNA samples: 16 larvae, 2 male brains, 1 male gonad, 2 female brains and 2 female gonads were sequenced at the Crown Genomics institute of the Nancy and Stephen Grand Israel National Center for Personalized Medicine (INCPM), Weizmann Institute of Science, Israel. Library preparations were done at the INCPM. In brief, 500 ng of total RNA were fragmented, reverse-transcribed and subjected to second-strand cDNA synthesis. Following end repair, adenine base addition, adapter ligation and PCR amplification, sequencing-libraries were created and evaluated by Qubit (Thermo Fisher Scientific, Waltham, MA, United States) and TapeStation (Agilent technologies, Inc. Santa Clara, CA, United States).

Sequencing libraries were constructed with barcodes to allow multiplexing of 24 samples in a single lane. Paired-end sequencing was conducted on an Illumina Nextseq500 platform with 150 bp paired-end reads. The data obtained was deposited in the sequence read archive (ENA bioproject accession no: PRJEB34342).

### Transcriptome Assembly

Raw reads were quality tested using FastQC^4^ and trimmed with Cutadapt (Martin, 2011). Trinity was used to perform *de novo* assembly following the Trinity pipeline for paired-reads assembly using default parameters (Grabherr et al., 2011). To maximize the assembly coverage for rare transcripts, a combination of all data sets was used including larvae, gonads and brains of both sexes. To assess the read content of the transcriptome assembly, Bowtie2 was used to align the reads of a single larva back to the assembled transcriptome (Langmead and Salzberg, 2012).

### Differential Expression Analysis and Functional Annotation

Following the Trinity pipeline, each sequencing library was aligned to the *de novo M. cephalus* assembled transcriptome by Bowtie (Langmead et al., 2009) and abundance estimation was done using RESM (Li and Dewey, 2011). Analysis of differentially expressed (DE) transcripts between male and female tissues and larvae sex-groups was performed in edgeR following the Trinity pipeline with a *p*-value of <0.01. Annotation was done using Trinotate (Bryant et al., 2017) following the recommended pipeline including Transdecoder for Open Reading Frames (ORFs) prediction. NCBI BLAST + (2.8.1) was used to query against SwissProt, Kyoto Encyclopedia of Genes and Genomes (KEGG), GO (Gene Ontology), and EggNog databases using BLASTX with an E-value cut-off set to 10-5 and BLASTP with an *E*-value cut-off set to 10-3. In addition, *O. niloticus* protein database was downloaded from NCBI and queried with similar *E*-value cut-offs.

### Assignment of Larvae Into Sex-Groups for Gene Expression

As the sexual phenotype of each individual will not be known until the fingerlings reach maturity at approximately 2–3 years of age, larvae groups were assigned into two putative sex-groups following segregation of paternal haplotypes, based on XY/XX sex-determination system (Dor et al., 2016).

### Mapping of Sex-Biased DE Transcripts to the Orthologous tLGs

Sex-biased DE transcripts of larvae, mature brains and mature gonads were queried against *O. niloticus* genome using GAP5 (Bonfield and Whitwham, 2010). The sum of hits per tLG was calculated. The relative abundance (RAi) of *M. cephalus* sex-biased DE transcripts, in the *i*th orthologous tLG, was calculated as the fraction from total sex-biased DE transcripts and weighted by the respective fraction of number of genes in the *i*th tLG from total, as follows:

(1) RAi=DEi×Gt/(DEt×Gi)

Where DEi is the number of sex biased *M. cephalus* DE transcripts localized to the *i*th tLG (*i* = 1–23). DEt is the total number of sex biased *M. cephalus* DE transcripts. Gi is the number of genes in the *i*th tLG (*i* = 1–23). Gt is the total number of genes in all tLGs.

## Results

### Comparative Mapping of the SDR of *M. cephalus*

**FIGURE 1:**
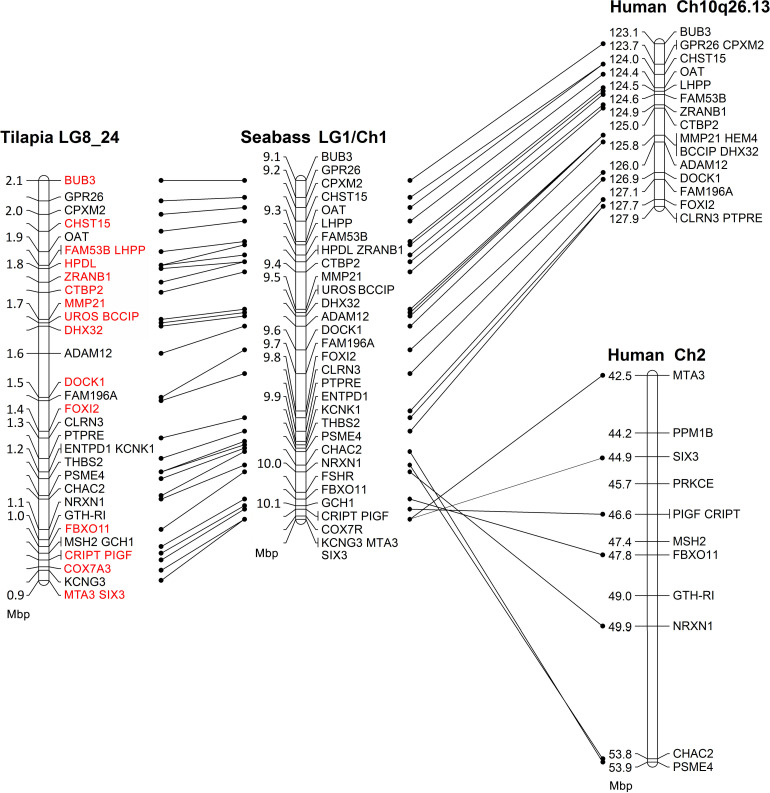
Comparative mapping of the sex determining genomic region between tilapia, seabass, and human. Red font annotates genes whose *M. cephalus* orthologs were assembled using male and female deep DNA sequencing data.

Comparative mapping of the SDR between tilapia, seabass and human is presented in Figure 1. The syntenic tLG8 for the SDR in *M. cephalus* contains 27 genes of which 26 were in synteny with *D. labrax* LG1. Only MSH2 was found in *O. niloticus* but not in *D. labrax* LG1. The syntenic genes were also conserved in human chromosomes 2 and 10. Ten orthologous genes in human with putative function on SD are presented in the Supplementary Table S2. Nineteen genes on tLG8 that are colored in red were assembled from *M. cephalus* male and female DNA sequencing data.

**FIGURE 2:**
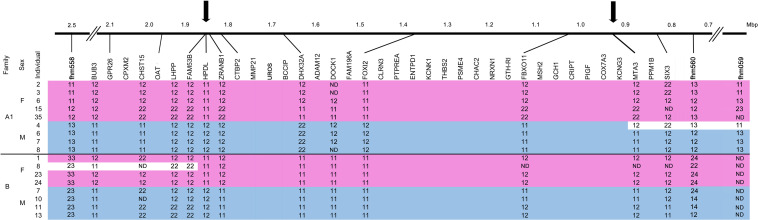
Refinement of the *M. cephalus* sex-determining region (SDR). Determination of boundaries of the *M. cephalus* SDR on the orthologous tLG8 based on genotyping of SNPs in 12 genes for selected 17 individuals of two families. For SNPs, the two homozygous genotypes are denoted “11” and “22” and the heterozygous is denoted as “12.” Additional genotypes for microsatellite markers with multiple alleles (1–3), flanking this region were also included (fhm markers, Dor et al., 2016). The genotypes’ segments corresponding to sex are shaded with pink and blue, for females and males, respectively. Inferred recombinant events within families, based on change of genotypes of multiple adjacent markers into the opposing sex pattern are shaded in white. ND represents “not determined” genotypes. The refined SDR is delimited by arrows and sized by Mbp units.

### Female Genome Sequencing and Assembly

Genome sequencing of *M. cephalus* produced a total of 301 million reads that were assembled into 4,505 scaffolds with an average length of 972 bp. The longest scaffold was of 24,439 bp. Most of the genome remained in a singleton state thus total number of scaffolds and singletons was 6,742,033 with average length of 151 bp (N50 = 410 bp). The estimated *M. cephalus* genome size was ∼1.02 Gbp and the genome coverage was 45-fold.

### Linkage and Physical Mapping of the SDR

A single representative marker genotype is shown for each of the 12 genes, along with other genes in their assembled positions but without genotypes (Figure 2). Associations between all 12 genes and sex were demonstrated for at least one of the two analyzed families. Recombination events were inferred from individuals that displayed the opposite sex genotype pattern within family for multiple adjacent markers (shaded in white). Male #4 in family A1 displayed the female pattern of genotypes for a stretch of four adjacent markers, whereas female #8 in family B displayed the male pattern of genotypes for three adjacent markers on the other end of the SDR. All other genotypes were in concordance to male and female patterns. Thus, the margin sites of recombination in both ends, represented by arrows, delimit the SDR in the orthologous tLG8 to less than 1 Mbp, between 0.96–1.85 Mbp, similar to the respective syntenic regions of *D. labrax* (9.4–10.15 Mbp), *P.reticulata* and O. *latipes* (Supplementary Table S3). The wild-type (WT) population of *M. cephalus* was analyzed for polymorphism in three selected genes (*FAM53*, *HPDL*, and *ZRANB1*), but surprisingly none of the genes were significantly associated with sex.

### Transcriptome Assembly

Paired-end RNA sequencing after adapters trimming and quality test yielded between 19.5 and 24.5 million reads per sample. One larva sample (C08) presented poor quality results and therefore was removed from the analysis. Eight libraries, with a total number of 176,016,446 reads, were used for the *de novo* transcriptome assembly representing different tissues, sex and developmental stage in order to build a comprehensive reference transcriptome. A total of 38,1457,653 bases were assembled using Trinity yielding 390,075 Trinity transcripts and a total of 282,328 Trinity “genes.” Average contig length was 978 bp and N50 was of 2,146 bp. For assessing the coverage efficiency of this transcriptome assembly, we mapped back the RNA-seq reads of a single larva onto the Trinity transcripts. This provided an overall alignment rate of 99.13% of its reads. The assembled transcriptome including annotations is available at http://cowry.agri.huji.ac.il.

### Assignment of Larvae Into Sex-Groups for Gene Expression

For parenthood analysis, six markers with 3–4 alleles each were genotyped for all 45 larvae at 28 dph and their potential parents (Supplementary Table S4). Potential male 1 (PM1) was excluded for at least 2 genetic markers for each of 44 larvae, and for a single marker in one individual. Potential male 2 (PM2) was excluded for at least 3 genetic markers for each of the 45 larvae. Thus, all 45 larvae were verified as progeny of a single male (PM3) out of the three males that were bred with a single female. Four haplotype groups were deduced with 8–14 samples per group (Supplementary Table S5). Thus, larvae were assigned into two putative sex-groups following segregation of paternal haplotypes, based on XY/XX sex-determination system (Dor et al., 2016).

### Differentially-Expressed (DE) Transcripts Analysis

**FIGURE 3:**
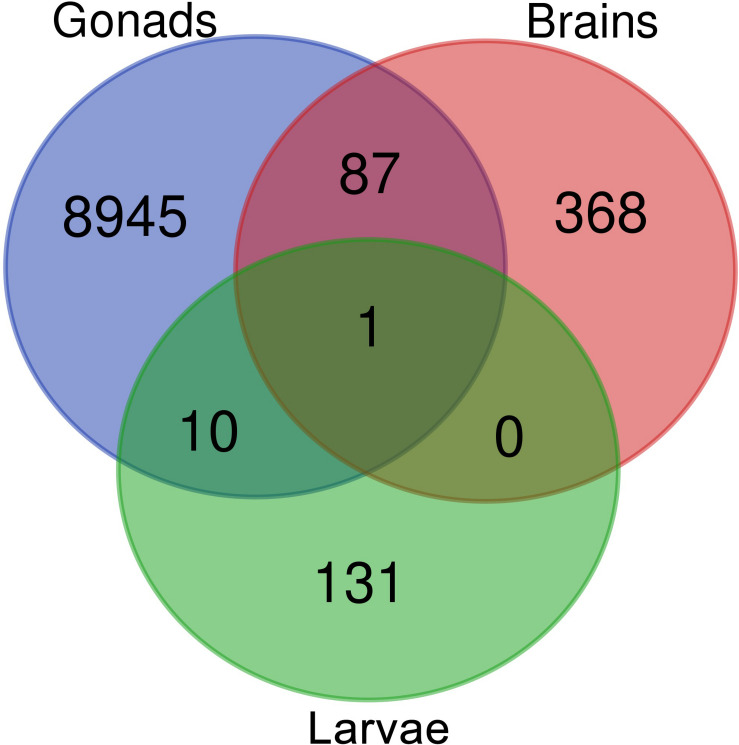
Venn diagram displaying the comparison of sex-biased annotated differentially expressed transcripts in tilapia from *M. cephalus* gonads, brains and larvae.

**FIGURE 4:**
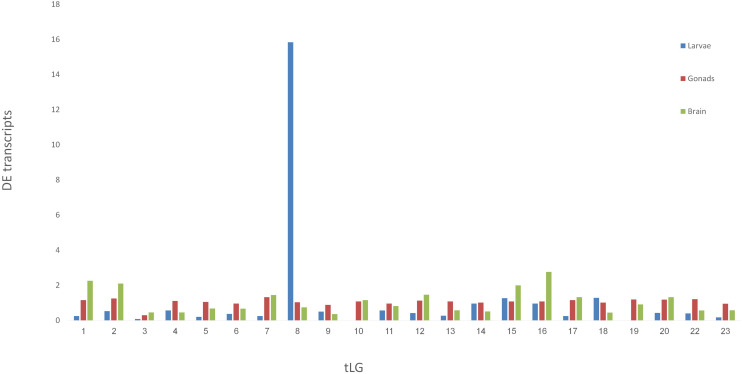
Mapping the *M. cephalus* sex-biased differentially-expressed genes (DE) in larvae to the orthologous tLGs. Larvae at 28 dph were assigned into two sex-groups following segregation of paternal haplotypes for markers in the sex determination region, based on XX/XY sex-determination system. The relative abundance of *M. cephalus* sex-biased DE transcripts, in each of the orthologous tLG, was calculated as the fraction from total of DE transcripts and weighted by the respective fraction of number of genes in tLG from total.

Venn diagram of sex-biased DE hits in tilapia from *M. cephalus* gonads, brain and larvae is presented in Figure 3. A total of 142 transcripts in larvae were sex-biased DE with 67 upregulated in group A and 75 in group B (*p* < 0.01). 24 Trinity contigs had no significant BLAST hits against vertebrate genomes, probably representing non-conserved sequences that are typical of untranslated regions (UTRs). Thus, 118 transcripts were annotated to 107 genes. The distribution of their locations in tLGs was determined. A total of 51 annotated DE transcripts (48%) (Supplementary Table S9), were mapped to tLG8, which harbors the SDR in *O. niloticus*, whereas all other transcripts were scattered in the 22 remaining tLGs (Figure 4). The relative abundance of annotated transcripts in larvae, which takes into account the number of genes in tLG, varied from 0.1 to 1.3 for the different tLGs, as compared to 15.8 for tLG8. This dozens-fold enrichment of sex-biased DE genes indicates that in larvae most of the sex-biased genes are transcribed in tLG8 that harbors the SDR (Figure 4). Of the 27 genes in the SD critical region three were DE displaying 11% enrichment: Dedicator of cytokinesis 1 (*DOCK1*), DEAH-box helicase 32 (*DHX32*), and *CDKN1A* Interacting Protein (*BCCIP*).

To test the validity of the XX/XY SD system for assignment of larvae into sex-groups, an alternative tentative assignment based on segregation of maternal haplotypes following the ZW/ZZ SD system was performed. Furthermore, larvae were randomly assigned into “sex-groups” to form a “negative control” for the comparison of sex-biased DE transcripts (Table 1). There were 107 sex-biased DE annotated transcripts when sex-groups were assigned following segregation of paternal haplotypes as compared to 44 by segregation of maternal haplotypes, and 38 by random segregation (Table 1). However, there was a significant enrichment of annotated transcripts that were mapped to tLG8 vs other tLGs, of 48 and 57% for both SD scenarios of XY and WZ, respectively, as compared to 5% of the negative control (Table 1). In contrast, of the sex-biased transcripts that were found in mature gonads (9043) and brains (456), only 350 (3.5%) and 13 (3%) of the transcripts, respectively, were mapped to tLG8; which is similar to the randomly expected proportions mapped to other tLGs (Figure 4), indicating lack of DE enrichment in tLG8 of adult tissues. The distribution of the number of DE transcripts was similar in both sex-groups.

**TABLE 1:** Mapping of sex-biased differentially expressed transcripts to tLG8 vs other tLGs.

	Mapping of sex-biased DE genes		
SD-system^1^	tLG8^2^	Other tLGs	All tLGs
XX/XY	51 (48%)	56	107
ZW/ZZ	25 (57%)	19	44
Control	2(5%)	36	38
Total	78	111	189

*^1^As sex may not be determined at early age, larvae were assigned into two sex-groups following segregation of paternal or maternal haplotypes, based on XX/XY or ZW/ZZ SD-systems, respectively. Random assignment of larvae was used as control. Chi-Squared test of mapping results was significant (*p* < 2.0 × 10^–8^). ^2^Enrichment of mapping sex-biased DE genes to tLG8 vs all tLGs is expressed in percent and presented in bracket.*

Ten transcripts were sex-biased DE in both larvae and mature gonads (Figure 3 and Supplementary Table S7). In addition, 87 transcripts were sex-biased DE in both adult’s brains and gonads, of which 65 were upregulated in females’ brains and gonads. The annotated genes are presented in Supplementary Table S8. These include Disabled-2 (*DAB2*) gene, which has been implicated in reproduction and is regulated by another DE: AKT Serine/Threonine Kinase 2 (*AKT2*). A single transcript was sex-biased DE in larvae, brains and gonads, and it was identified as ADP ribosylation factor 1 (*ARF1*). This transcript was elevated in larvae group B and females’ brains and gonads. *ARF1* was assembled and mapped to tLG18 and its polymorphism was not associated with sex in gray mullet. A second *ARF1* splice isoform was upregulated in testis and in larvae group A. Both *ARF1* isoforms were aligned to the non-translated 3′ region.

### Gene Ontology (GO) Enrichment Analysis

A total of 142,516 transcripts were annotated using queries against SwissProt and Pfam databases (Trinotate report). Transdecoder predicted 142,744 ORFs and clustering by CD-HIT-EST resulted in 82,997 sequence clusters. GO enrichment analysis of sex-biased DE transcripts in larvae and brain produced non-significant results. However, 36 (6%) significantly enriched GO terms were obtained for female gonad, as compared to 682 (36%) in male gonad with *p*-value < 0.05 after FDR correction (Supplementary Table S9).

## Discussion

Conserved synteny among different Teleostei species is useful when analyzing a non-model fish that does not have a reference genome (Nadeau, 1989; Rondeau, 2017). Previously, we presented a conserved synteny between *O. niloticus* (tLG8) and LG9 of *M. cephalus* (Dor et al., 2016). In the current study, comparative mapping for the SDR showed a conserved synteny between LG1 of *D. labrax* and tLG8 (Figure 1). Thus, in the absence of a reference genome for gray mullet, this synteny with tilapia, seabass and human (Chromosomes 2 and 10) was used to order the assembled *M. cephalus* genomic sequence of a single female within the SDR. This assembly was compared to its previously sequenced full-sib male. The resulting assembly of 19 genes was followed by polymorphism detection in 12 genes. Association with sex for markers along the SDR was significant for at least one of the two analyzed families, but not in WT population. Thus, the genetic markers for sex were not in population-wide linkage disequilibrium. Nevertheless, analysis of individual recombinants following Eshel et al. (2012), indicated the boundaries of the sex region to ∼0.9 Mbp in the orthologous tLG8 harboring 27 genes, and ∼0.7 Mbp in the orthologous *D. labrax*, narrowing down the SDR by two-fold as compared to the previous mapping (Dor et al., 2016). Syntenic relationships between mullet and tilapia genomes indicate similar gene order between these species, but some changes in genes’ location may still occur.

Sex-biased DE transcripts were found in larvae (142) and adults’ gonad (9043) and brain (456) (Figure 3). GO enrichment analysis of sex-biased DE genes was effective only in gonads, yielding 36 and 682 significant GO in females and males, respectively (Supplementary Table S9). Ovaries undergo physiological changes along time and display larger variation than testis, which may explain the enhanced enrichment of GO terms in testis. The 10 sex-biased DE transcripts in both larvae and mature gonads (Figure 3) included *DAB2*, which has been implicated in the regulation of the MII stage oocyte formation and other crucial processes for porcine reproductive competence (Budna et al., 2017). In addition, *DAB2* plays an essential role during TGF β-mediated epithelial to mesenchymal transition (EMT) effecting autophagy and apoptosis processes (Chaudhury et al., 2010; Jiang et al., 2016). Another DE gene, *AKT2*, is involved in promoting EMT and inducing *DAB2* gene expression (Chaudhury et al., 2010).

Based on gonadal histology, distinguished sex differentiation of the flathead gray mullet emerges at age of 12 months although the genetic factors affecting primary SD are expressed at a much earlier age (Chang et al., 1995). For example, in tilapias which reach maturity at 3 months, SD is initiated at 2 to 10 days post fertilization (dpf) (Ijiri et al., 2008; Kobayashi et al., 2008; Eshel et al., 2014). *IGFBP2* and *KCTD5* genes were sex-biased DE in trout between 15 and 90 dpf (Hale et al., 2011). We studied expression of flathead gray mullet larvae of both sexes at 28 dph, which is the earliest age that enables minimal RNA extraction of individual larva, although SD factors may be expressed earlier.

Several SD related genes are found among annotated transcripts that are upregulated in adult females’ brains and gonads. Only a single transcript annotated to the gene *ARF1* was elevated in females’ brains and gonads and in larvae group B. In mice, *ARF1* was suggested to play an essential role in regulating asymmetric cell division in female meiosis through a proposed *ARF1*-*MAPK* pathway (Wang et al., 2009). In addition, *ARF1* was found to be upregulated in females’ brains of *Macaca fascicularis* (Reinius, 2006). Interestingly, a second *ARF1* splice isoform was commonly upregulated in testis and larvae of sex-group A, which suggests sexual regulation of *ARF1* splicing in *M. cephalus*. However, the identified polymorphism in this gene was not associated with sex in gray mullet.

There were 2.4-fold more sex-biased DE genes (107 vs 44) when assigning larvae to sex-groups by paternal (XY) rather than by maternal (ZW) segregation of haplotypes, lending further support to our previous finding for males being heterogametic for this SDR (Dor et al., 2016). A significant enrichment of sex-biased DE genes that were mapped to tLG8 vs other tLGs, was evident when assigning SD groups by the paternal (51 genes, 48%) or maternal (25 genes, 57%) paths (Table 1 and Figure 4). Only two genes Homeobox-and-Leucine-Zipper-Encoding (HOMEZ) and Leucine-Rich-Repeat, Ig-like-and-Transmembrane-Domains 1 (*LRIT1*) were sex-biased DE in both SD scenarios (Supplementary Table S6). Trans-generation imprinting may be responsible for enrichment of different sex-biased DE genes localized to tLG8, based on different meiosis in the parental generation (Bošković and Rando, 2018). Furthermore, 23 of the 107 sex-biased DEs genes were tagged, based on their consistent expression among all individuals of one sex-group and absence of expression in all individuals of the other sex-group i.e., sex-specific. Twenty-two of the 23 genes were positioned in tLG8, indicating 96% enrichment. Leder et al. (2010) have reported a highly significant proportion (23%) of sex-biased DE genes that are concentrated on chromosome 19, corresponding to the sex chromosomes in three-spined stickleback (*Gasterosteus aculeatus*). Thus, the same pattern of enrichment has been previously observed with a significant bias toward female DE genes. In our study, the level of enrichment was much higher with no bias toward any of the sex-groups. Random DE genes in the control “sex-groups” were scattered across all tLGs, with 5% in tLG8 as compared to the expected 4.3% assuming random segregation in 23 tLGs (1/23). Thus, they were apparently “false positives” (Table 1). We propose that most falsely discovered DE genes in the comparison of the two sex-groups of larvae should be mapped to different tLGs, rather than to tLG8. Thus, we hypothesize that the 51 genes, that are orthologs of tLG8 genes, constitute a unique enriched cluster of positional genes that has a major role in SD of *M. cephalus*.

Of the 51 sex-biased DE genes in larvae, 12 were reported in association with SD through a literature survey (Supplementary Table S6). *EP300* has been suggested to have a role in testis determination mediated by control of histone acetylation at the *Sry* locus (Carré et al., 2018). Interestingly, the SDR gene *CTBP2* has been found to closely interact with *EP300* (Bajpe et al., 2013), although it was not a sex-biased DE. However, sex-biased DE genes may also be a result of regional imprinting on sex chromosomes (dosage compensation). Extensive expression differences are encountered between sex chromosomes transmission through male or female germlines as a result of epigenetic modification of the Y chromosome, or inactivation of one of the two X chromosomes (Golic et al., 1998; Lemos et al., 2014). Imprinting in Drosophila has been detected by transmission of visible chromosomal elements through male gametes, such as P-element insertions on the Y chromosome (Haller and Woodruff, 2000). Different copy numbers of X chromosome between *D. melanogaster* males and females are compensated by increasing transcription of a single X chromosome in males (Baker et al., 1994). Conversely, male hypermethylated (MHM) genes that have been localized to a region on the Z chromosome in birds are expressed only in a single Z chromosome of females (Caetano et al., 2014). In fly, chicken and mammals, significant morphological differences between sex chromosomes are encountered indicating significant differences in their DNA content (Naurin et al., 2010).

In *M. cephalus* no morphological differences between DNA of both sexes were detected, but appearance of 48% sex-biased DE genes on the short tLG8 indicates the existence of regional imprinting on mullet sex chromosomes. Moreover, in fly and mammals there are reported genetic factors (MSL complex and XIST gene) that localize on sex chromosomes and initiate the mechanism of dosage compensation (Legube et al., 2006; Keller and Akhtar, 2015).

In the present study, we detected three sex-biased DE genes (*EP300*, *MSL1*, and *SCML2*) that may play a role in regional imprinting on sex chromosomes. While *EP300* and *SCML2* are factors involved in transcription regulation via modification of histones, *MSL1* is a major factor of fly MSL complex. Both *EP300* and *MSL1* genes were located on tLG8 at positions of 11 and 6 Mbp, respectively. *BCCIP*, *DHX32A*, and *DOCK1* displayed the same pattern of sex-biased DE in larvae and localized to the orthologous SD critical region of tLG8. Only *DOCK1* was expressed among all larvae of one sex-group with no expression in all larvae of the other sex-group. While *BCCIP* was not analyzed for association with sex, because of a lack of polymorphism, both *DOCK1* and *DHX32A* genes were associated with sex along with 10 other genes in the critical SDR (Figure 2). *DOCK1* has been suggested to be involved in polycystic ovarian syndrome, due to its downregulated expression in polycystic ovary (Ubba et al., 2017). DEAD/H-box proteins are involved in various aspects of RNA metabolism, including sperm-oocyte switch in *C. elegans* (Konishi et al., 2008) and spermatogenesis regulation in mice (Dufau and Tsai-Morris, 2007). *DHX32A* was the only DExD/H-box RNA helicase gene that has been found to have two copies in most of the teleost species, and is highly expressed in channel catfish females (Tian et al., 2017). *BCCIP* is an important BRCA2 cofactor in tumor suppression, which has been implicated in many cellular processes; including telomere maintenance, recombination and damage repair, embryonic development and genomic stability (Liu et al., 2013). It is upregulated in male rainbow trout embryos (Hale et al., 2011), and has been suggested to have a key role in neural development (Huang et al., 2012). In addition, *GTH-RI* i.e., Follicle Stimulating Hormone Receptor (*FSHR*) is mapped to the SDR. This gene functions in gonad development, and mutations in the gene cause ovarian dysgenesis (Aittomaki et al., 1995). However, *FSHR* expression was low in the analyzed larvae (annotated in our Mullet Database as gene_id: TRINITY_DN4978_c0_g2). Yet, these four genes are candidates by putative position and function for master key regulators of SD.

## Conclusion

**FIGURE 5:**
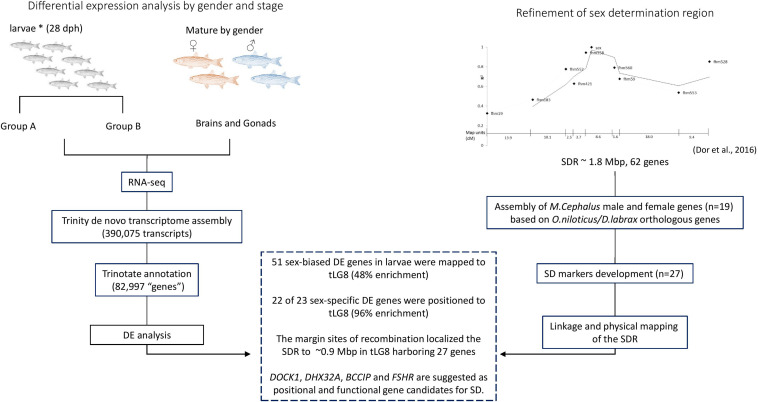
Schematic representation of the workflow and integrative results.

The schematic representation of the workflow of this study and the integrative results are presented in Figure 5. The main tools being used to study the SDR were comparative mapping, polymorphism detection, linkage mapping, RNA-seq, literature survey on function of genes and analysis of marker-association with sex. The main findings were: (1) 48% of the sex-biased DE annotated transcripts in larvae were localized to tLG8, which harbors the SDR, indicating positional enrichment of genes related to SD and differentiation; (2) 22 of the 23 genes with sex-specific expression were positioned to tLG8 (96% enrichment); (3) The SDR was narrowed by two-fold, down to less than 1 Mbp in the orthologous tLG8 harboring 27 genes; and (4): *BCCIP*, *DHX32A*, *DOCK1*, and *FSHR* (*GTH-RI*) are suggested as positional and functional gene candidates for master key regulators of SD. Polymorphism in one of these genes displaying population-wide association with sex, would indicate the identity of the SD regulator.

## Data Availability Statement

Raw sequence data of sequenced female are available through the European Nucleotide Archive (ENA) database accession number ERX3552876. The final transcriptome assembly is locally hosted at our server and available for browsing at http://cowry.agri.huji.ac.il. Raw sequence data are available through the European Nucleotide Archive (ENA) database under project’s accession number PRJEB34342.

## Ethics Statement

The animal study was reviewed and approved by AEEC in accordance with the requirements of the Prevention of Cruelty to Animals (Animal Experimentation) 1994 Act.

## Author Contributions

IA and HR reared and sampled the specimens used in the study. ES assembled the female’s sequence data and constructed the initial assembly, contributed to the bioinformatics analysis, and advised on the project. JW contributed to the statistical analysis. LD assembled the candidate genes and RNA-seq data. LD, MR, AS, and AC contributed to the experimental design, the candidate genes, RNA-seq analysis, and drafting the manuscript. MR supervised the study. All authors contributed to the article and approved the submitted version.

## Conflict of Interest

The authors declare that the research was conducted in the absence of any commercial or financial relationships that could be construed as a potential conflict of interest.

## Footnotes

1 www.fishbase.de

2 http://sourceforge.net/projects/soapdenovo2

3 http://manned.org/TrimmomaticPE

4 http://www.bioinformatics.babraham.ac.uk/projects/fastqc/

## Supplementary Material

TABLE S1 Primers for SD markers.

TABLE S2 SD related genes on the orthologous SDR in Human genome.

TABLE S3 Comparison of orthologous SDR of four fish species based on order of genes in O. niloticus (tLG8, 10564505–11503641 bp).

TABLE S4 Parenthood identification summary based on the “exclusion principle.”

TABLE S5 Larvae assignment to putative sex groups by paternal haplotypes.

TABLE S6 Sex-biased differentially expressed genes in larvae which were localized to tLG8.

TABLE S7 Sex-biased differentially expressed genes in both larvae and gonads.

TABLE S8 Sex-biased differentially expressed genes in both brains and gonads.

TABLE S9 Enrichment of Gene Ontology (GO) terms for sets of sex-biased differentially expressed genes in gonads.
