# Supplementary material for: Preferential Mapping of Sex-Biased Differentially-Expressed Genes of Larvae to the Sex-Determining Region of Flathead Grey Mullet (Mugil cephalus)
Source: Front Genet. 2020 Aug 21;11:839. doi: 10.3389/fgene.2020.00839 (PMC7472742; doi:10.3389/fgene.2020.00839)
Supplement: TABLE S1 — Primers for SD markers. [file Data_Sheet_1.pdf]

Table S1. Primers for SD markers

Marker	Forward primer	Reverse primers
BUB3int5	GCCATCTCATTTCACAGTGC	GGTCCCAGATGTTCACGAAG
CHST15ex4	CTTTACCGCATCCTGGAGAC	CAACGCCTGGGTGTATTTCT
CHST15ex8	GCTTGCATCCTTTCTGGGTA	GGGACAAGAAGGGTCTCCTC
DHX32	CTTTGGTATTTTGGGGCAGA	GGAAGGTCAGGTGCCAGTT
FAM53	TGGACTATTACGTGCATTCCAG	GCCACAGCTGCGCTATACA
FOXI2	GGACCCCAACTGTGAGAAGA	AGTTCACGCTGGACACGAAG
FXBO11ex2	AAGGGACCAGTGATTTGGTG	CCAGCAGACAAGGGAAAATC
FXBO11ex8	TCAACTGCAGTCCGAACATC	CATGGCCACTATGTAGGAGGA
HPDLex1	TACAGACACGCCAACTCTGC	CCACAATGGACTTCATCACG
HPDLex2	TTACGGCCATGGAGTACTGG	1: GACCTGGTTCCTGCCTGA
		2: AATCTGGTTCCTGCCTGAAA
HPDLex5A	GACATGTCGCCACGTCTTC	AGGTTCAACGATATAAATACAAAACA
HPDLex5B	ATGCCGCCATGTCTTCAG	CCGTTTAACGATATAAATACAAAACA
LHPP	TGTTCCCATGCAAGAATTAGC	TTCATGTTGCGGTAGGAAAA
MMP21ex2	ATCGGAGAAGGCTACAGCAG	CTTTGTCTCGACCGACCTGT
MMP21ex3	CCTGCTTCATTGCAAGTTTTT	GAAATGCTCGTCGTCATCAA
MMP21ex7	ACGCCGCCTACTTCTCCTAC	CAGGTCTGATCCACCTCCTG
MTA3	AGGAAGGAAACAACGCCTTT	CAATTTCACACTTGCCATGC
MTA3A	TAGAGGGTGCTGTTTGTTGC	GCTCGTTAACAACGCTGAAA
MTA3B	AGTGCTGCAGTCATTCATGC	TAGCAGCTCTCGCAGGGTCT
SIX3	GCGTCTCATCTTGCCTCACT	GAAACTTCCCGTGTGGTAGG
ZRANBex1A	CATCATCACGGAGGAACCTT	CGAGGCCAGTTCAGATAGGT
ZRANBex1B	ATAGCCGAGCTTGCCAATAA	GGGCAAGTTCTGGTAAGTGC
ZRANBex9	GAGAAGCTGCTGAGGGAGTG	AGTTGCCCTGACTCACTCGT
