# Supplementary material for: Preferential Mapping of Sex-Biased Differentially-Expressed Genes of Larvae to the Sex-Determining Region of Flathead Grey Mullet (Mugil cephalus)
Source: Front Genet. 2020 Aug 21;11:839. doi: 10.3389/fgene.2020.00839 (PMC7472742; doi:10.3389/fgene.2020.00839)
Supplement: TABLE S2 — SD related genes on the orthologous SDR in Human genome. [file Data_Sheet_2.pdf]

Table S2. SD related genes on the orthologous SDR in Human genome

Human chromosome location (bp)		Gene annotation	Gene Symbol	Reference
Chr2	47045538	Sperm-tail PG-rich repeat containing 4	*STPG4*	
	48314637	Forkhead box N2	*FOXN2*	Chen et al., 2017
	48686775	Luteinizing hormone/choriogonadotropin receptor	*LHCGR*	
	48962157	Follicle stimulating hormone receptor	*FSHR*	
	53864067	Proteasome activator subunit 4	*PSME4*	
Chr10	123154277	BUB3, mitotic checkpoint protein	*BUB3*	Li et al., 2009
	123706207	Carboxypeptidase X, M14 family member 2	*CPXM2*	Rajpathak et al., 2014
	125576522	Testis expressed 36	*TEX36*	
	125836337	DEAH-box helicase 32	*DHX32*	Tian et al., 2017
	126905409	Dedicator of cytokinesis 1	*DOCK1*	Yatsenko et al., 2009
