# Supplementary material for: Preferential Mapping of Sex-Biased Differentially-Expressed Genes of Larvae to the Sex-Determining Region of Flathead Grey Mullet (Mugil cephalus)
Source: Front Genet. 2020 Aug 21;11:839. doi: 10.3389/fgene.2020.00839 (PMC7472742; doi:10.3389/fgene.2020.00839)
Supplement: TABLE S6 — Sex-biased differentially expressed genes in larvae which were localized to tLG8. [file Data_Sheet_6.pdf]

**Table S6.** Sex-biased differentially-expressed genes in larvae which were localized to tLG8.

| Elevated in group | Annotation | gene | FDR | SD related Reference[1] |
|---|---|---|---|---|
| A | Dedicator of cytokinesis 1 | *DOCK1* | 3.0E-05 | |
| | DEAH-box helicase 32 (putative) | *DHX32* | 2.6E-24 | Tian et al., 2017 |
| | BRCA2 and CDKN1A interacting protein | *BCCIP* | 2.6E-24 | Hale et al., 2011 |
| | Ornithine aminotransferase | *OAT* | 9.3E-35 | Hale et al., 2011 |
| | BUB3 mitotic checkpoint protein | *BUB3* | 4.3E-04 | Leelatanawit et al., 2009 |
| | Poly(ADP-ribose) glycohydrolase | *PARG* | 1.2E-11 | |
| | Solute carrier family 18 member A3 | *SLC18A3B* | 2.7E-13 | |
| | Ataxin 2 like | *ATXN2L* | 1.1E-04 | |
| | Ring finger protein 40 | *RNF40* | 4.7E-19 | |
| | Marker of proliferation Ki-67 | *MKI67* | 2.1E-21 | |
| | Integrin subunit alpha 3 | *ITGA3* | 3.6E-05 | |
| | Alanyl-tRNA synthetase domain containing 1 | *AARSD1* | 4.1E-48 | |
| | E1A binding protein P300 | *EP300* | 1.2E-14 | Liu., 2015 |
| | Corticotropin releasing hormone receptor 1 | *CRHR1* | 1.9E-05 | |
| | MSL complex subunit 1 | *MSL1* | 1.6E-06 | Shi et al., 2015 |
| | Transmembrane protein 106B | *TMEM106B* | 1.3E-13 | |
| | Leucine rich repeat containing 17 like | *LRRC17L* | 7.8E-05 | |
| | Heparan sulfate-glucosamine 3-sulfotransferase 3B1 | *HS3ST3B1* | 9.6E-10 | |
| B | SEC24 homolog b, COPII coat complex component like | *SEC24B like* | 2.4E-03 | |
| | Leucine rich repeat, ig-like and transmembrane domains 1 | *LRIT1* | 4.1E-10 | |
| | DNA cross-link repair 1A | *DCLRE1A* | 2.1E-03 | |
| | TLC domain containing 3B | *TLCD3B* | 2.4E-03 | |
| | Carbonic anhydrase 4 like | *CA4L* | 2.7E-05 | |
| | Ankyrin repeat and fibronectin type III domain containing 1 | *ANKFN1* | 2.6E-11 | |
| | CCZ1 homolog, vacuolar protein trafficking and biogenesis associated | *CCZ1* | 6.0E-03 | |
| | Neuronal pentraxin 2 like | *NPTX2L* | 7.7E-06 | Elzaiat et al., 2014 |
| | Methionine sulfoxide reductase B1 like | *MSRB1L* | 4.3E-10 | |
| | MTOR associated protein, LST8 homolog | *MLST8* | 1.9E-12 | |
| | Aconitase 2 | *ACO2* | 5.4E-25 | |
| | Potassium channel tetramerization domain containing 5 like | *KCTD5L* | 1.2E-06 | Hale et al., 2018 |
| | Hexosaminidase D | *HEXDC* | 3.4E-08 | |
| | 6-pyruvoyltetrahydropterin synthase like | *PTSL* | 2.9E-05 | |
| | ADP-ribose/CDP-alcohol diphosphatase, manganese dependent | *ADPRM* | 2.6E-17 | |
| | Potassium channel tetramerization domain containing 21 | *KCTD21* | 1.9E-04 | |
| | Ubiquitin conjugating enzyme E2 D4 like | *UBE2D4L* | 1.0E-20 | |

| | | | | |
|---|---|---|---|---|
| | RNA polymerase III subunit D | *POLR3DL* | 2.7E-03 | |
| | Microtubule associated protein tau like | *MAPTL* | 5.6E-06 | |
| | Transmembrane protein 150A | *TMEM150A* | 1.2E-42 | |
| | Thyroid hormone receptor alpha-B | *TR-alpha1* | 3.6E-03 | Leelatanawit et al., 2009 |
| | Dual specificity phosphatase 3 | *DUSP3* | 5.5E-05 | Hao et al., 2013 |
| | Sodium voltage-gated channel alpha subunit 4 | *SCN4A* | 3.6E-03 | |
| | G Protein subunit gamma transducin 2 | *GNGT2* | 6.3E-04 | |
| | Tripartite motif containing 8 | *TRIM8* | 1.1E-03 | |
| | ADP ribosylation factor like GTPase 3 | *ARL3* | 1.7E-12 | Wang et al., 2018 |
| Both[2] | Annexin A4 like | *ANXA4* | 5.7E-26 | |
| | ADP ribosylation factor 1 | *ARF1* | 9.1E-06 | Reinius 2006 |
| | Cerebellar degeneration related protein 2 like | *CDR2L* | 2.9E-05 | |
| | Dual specificity phosphatase and pro isomerase domain containing 1 | *DUPD1* | 1.3E-07 | |
| | NudE neurodevelopment protein 1 like 1 | *NDEL1* | 2.4E-09 | |
| | Thyrotrophic embryonic factor | *TEF like* | 9.6E-05 | |
| | WD repeat domain 90 | *WDR90* | 8. 8E-06 | |

[1]Publication in which the gene was sex-biased DE. [2]Different transcripts annotated to the same gene were upregulated in both larvae sex-groups
