# Supplementary material for: Preferential Mapping of Sex-Biased Differentially-Expressed Genes of Larvae to the Sex-Determining Region of Flathead Grey Mullet (Mugil cephalus)
Source: Front Genet. 2020 Aug 21;11:839. doi: 10.3389/fgene.2020.00839 (PMC7472742; doi:10.3389/fgene.2020.00839)
Supplement: TABLE S7 — Sex-biased differentially expressed genes in both larvae and gonads. [file Data_Sheet_7.pdf]

Table S7. Sex-biased differentially-expressed genes in both larvae and gonads

DE in larvae group	Gonads	
	Female	Male
A	WASP Family Member 1 (*WASF1*),<br>Ataxin 2 Like (*ATX2L*)	WD repeat domain 90 (*WDR90*),<br>AKT Serine/Threonine Kinase 2 (*AKT2*),<br>Obscurin Like 1(*OBSL1*),<br>ADP Ribosylation Factor 1 (*ARF1*) isoform 2
B	Neural cell adhesion molecule L1 like (*NCHL1*),<br>ADP Ribosylation Factor 1 (*ARF1*) isoform 1,<br>DAB Adaptor Protein 2 (*DAB2*)	family with sequence similarity 198 member A (*FAM198A*),<br>Microtubule Actin Crosslinking Factor 1 (*MACF1*)
