# Supplementary material for: Preferential Mapping of Sex-Biased Differentially-Expressed Genes of Larvae to the Sex-Determining Region of Flathead Grey Mullet (Mugil cephalus)
Source: Front Genet. 2020 Aug 21;11:839. doi: 10.3389/fgene.2020.00839 (PMC7472742; doi:10.3389/fgene.2020.00839)
Supplement: TABLE S8 — Sex-biased differentially expressed genes in both brains and gonads. [file Data_Sheet_8.pdf]

Table S8. Sex-biased differentially-expressed genes in both brains and gonads

DE in Sex	Gene annotation	Symbol
Male	Septin-8	*SEPT8*
	Lysine-specific demethylase 5B-B	*KDM5BB*
	Nucleolin	*NCL*
	Growth hormone-regulated TBC protein 1-A	*GRTP1A*
	Dihydrolipoyllysine-residue succinyltransferase component of 2-oxoglutarate dehydrogenase complex, mitochondrial	*DLST*
	MHC class II regulatory factor RFX1	*RFX1*
	Rab11 family-interacting protein 1	*RAB11FIP1*
	Interferon-induced very large GTPase 1	*GVIN1*
	Lysyl oxidase homolog 1	*LOXL1*
Female	Retinol binding protein 2	*RBP2*
	Dihydrolipoamide acetyltransferase component of pyruvate dehydrogense	*SUCB1*
	cAMP-responsive element-binding protein-like 2	*CRBL2*
	Histone H2A.Z	*H2AZ*
	Apoptotic chromatin condensation inducer 1	*ACIN1*
	Aryl hydrocarbon receptor nuclear translocator 2	*ARNT2*
	Small integral membrane protein 19	*SMIM19*
	Heat shock 70 kDa protein 4	*HSPA4*
	Coactosin-like protein	*COTL1*
	4F2 cell-surface antigen heavy chain	*SLC3A2*
	Sphingosine-1-phosphate phosphatase 1	*SGPP1*
	Serine incorporator 5	*SERINC5*
	Transcriptional regulator ATRX	*ATRX*
	Monocarboxylate transporter 1	*SLC16A1*
	1-acyl-sn-glycerol-3-phosphate acyltransferase gamma	*AGPAT3*
	Alpha-1B adrenergic receptor	*ADRA1B*
	Vacuolar protein sorting-associated protein 4A	*VPS4A*
	Saccharopine dehydrogenase-like oxidoreductase	*SCCPDH*

Vacuolar ATPase assembly integral membrane protein vma21	*VMA21*
GRIP and coiled-coil domain-containing protein 2	*GCC2*
Glyoxylate reductase/hydroxypyruvate reductase	*GRHPR*
Ubiquitin carboxyl-terminal hydrolase 19	*UBP19*
S-adenosylmethionine synthase isoform type-2	*MAT2A*
Apoptotic chromatin condensation inducer in the nucleus	*ACIN1*
RAB6A-GEF complex partner protein 2	*RGP1*
Pyruvate carboxylase, mitochondrial	*PC*
39S ribosomal protein L18, mitochondrial	*MRPL18*
KAT8 regulatory NSL complex subunit 1-like protein	*KANSL1L*
V-type proton ATPase subunit e 1	*ATP6V0E1*
Alpha-parvin	*PARVA*
Eukaryotic initiation factor 4A-I	*EIF4A1*
Septin-8-A	*SEPT8A*
E3 ubiquitin-protein ligase Mdm2	*MDM2*
ADP-ribosylation factor 4	*ARF4*
Ras-related protein Rab-35	*RAB35*
Mothers against decapentaplegic homolog 5	*SMAD5*
Long-chain-fatty-acid--CoA ligase 6	*ACSL6*
Apoptosis-stimulating of p53 protein 2	*TP53BP2*
P2X purinoceptor 7	*P2RX7*
Partitioning defective 3 homolog B	*PARD3B*
Transducin-like enhancer protein 1	*TLE1*
Teashirt homolog 3	*TSH3*
Serine/threonine-protein kinase 35	*STK35*
Gamma-taxilin	*TXLNG*
Kelch-like protein 12	*KLHL12*
Heat shock 70 kDa protein 13	*HSPA13*
Ubiquitin-conjugating enzyme E2 G2	*UBE2G2*
Arf-GAP with SH3 domain, ANK repeat and PH domain-containing protein 2	*ASAP2*

PDZ and LIM domain protein 7	*PDLIM7*
Protein arginine N-methyltransferase 7	*PRMT7*
PHD and RING finger domain-containing protein 1	*PHRF1*
Methionine aminopeptidase 2	*METAP2*
