# Supplementary material for: Preferential Mapping of Sex-Biased Differentially-Expressed Genes of Larvae to the Sex-Determining Region of Flathead Grey Mullet (Mugil cephalus)
Source: Front Genet. 2020 Aug 21;11:839. doi: 10.3389/fgene.2020.00839 (PMC7472742; doi:10.3389/fgene.2020.00839)
Supplement: TABLE S9 — Enrichment of Gene Ontology (GO) terms for sets of sex-biased differentially expressed genes in gonads. [file Data_Sheet_9.pdf]

Table S9. Enrichment of Gene Ontology (GO) terms for sets of sex-biased differentially-expressed genes in gonads.

A. Significantly enriched GO terms - 682 out of 1892 in male (p <0.05)

Selected GO Terms	DE (N)[1]	FDR[2]	Representative male DE genes (out of 2824)
Cell adhesion (GO:0007155, BP)	153 (590)	3.9E-15	Insulin-like growth factor-binding protein 7 (*IBP7*); Sorbin and SH3 domain-containing protein 1 (*SRBS1*); Filamin-binding LIM protein 1 (*FBLI1*); Peripheral myelin protein 22 (*PMP22*); Transforming protein RhoA (*RHOA*).
Regulation of locomotion (GO:0040012, BP)	162 (743)	1.6E-09	Bone morphogenetic protein 8B (*BMP8B*); Nuclear factor erythroid 2-related factor 2 (*NF2L2*); Matrix metalloproteinase-28 (*MMP28*); C-C motif chemokine (*CCL25*); Dual specificity protein phosphatase 1 (*DUS1*).
Regulation of cell motility (GO:2000145, BP)	150 (677)	2.7E-09	Nuclear factor erythroid 2-related factor 2 (*NF2L2*); Dispanin subfamily A member 2b (*DSA2B*); HLA class II histocompatibility antigen gamma chain (*HG2A*); Caveolae-associated protein 1 (*CAVN1*).
Cell motility (GO:0048870, BP)	148 (678)	7.0E-09	(*TGFB1*) Transforming growth factor beta-1 proprotein; StAR-related lipid transfer protein 13 (*STA13*); Receptor-type tyrosine-protein phosphatase O (*PTPRO*); Amyloid-beta A4 precursor protein-binding family B member 1 (*APBB1*); NACHT, LRR and PYD domains-containing protein 8 (*NALP8*).
Signal transduction (GO:0007165, BP)	465 (2770)	9.6E-09	Sorbin and SH3 domain-containing protein 1 (*SRBS1*); Cyclic AMP-dependent transcription factor ATF-4 (*ATF4*); 5'-AMP-activated protein kinase subunit gamma-3 (*AAKG3*); C-terminal-binding protein 1 (*CTBP1*); Regulator of G-protein signaling 1(*RGS1*); Potassium channel subfamily K member 10 (*KCNKA*).
Cilium movement (GO:0003341, BP)	17 (43)	7.6E-04	Cilia- and flagella-associated protein 206 (*CF206*); Intraflagellar transport protein 57 (*IFT57*); Tubulin polyglutamylase (*TTLL6*); Tektin-1

			(*TEKT1*); Coiled-coil domain-containing protein 103 (*CC103*).
Sperm part (GO:0097223, CC)	29 (108)	3.6E-03	Sperm-associated antigen 8 (*SPAG8*); Intraflagellar transport protein 81 (*IFT81*); Izumo sperm-egg fusion protein 1 (*IZUM1*); Spermatogenesis-associated protein 6 (*SPAT6*).
Sperm midpiece (GO:0097225, CC)	6 (11)	3.4E-02	Sperm flagellar protein 2 (*SPEF2*); cAMP-dependent protein kinase catalytic subunit beta (*KAPCB*); Intraflagellar transport protein 81 homolog (*IFT81*); Gamma-aminobutyric acid receptor-associated protein (*GBRAP*); Parkin coregulated gene protein homolog (*PACRG*)

B. Significantly enriched GO terms - 36 out of 622 in female (p <0.05)

Selected GO Terms	DE (N)	FDR	Representative female DE genes (out of 3095)
Egg coat formation (GO:0035803, BP)	12 (12)	5.9E-09	Zona pellucida sperm-binding protein 3 (*ZP3*); Sodium-dependent lysophosphatidylcholine symporter 1-A (*NLS1A*).
Regulation of fertilization (GO:0080154, BP)	18 (28)	6.4E-09	Tetratricopeptide repeat protein 32 (*TTC32*); High choriolytic enzyme 1 (*HCE1*); Zona pellucida sperm-binding protein 4 (*ZP4*); High choriolytic enzyme 2 (*HCE2*); Zona pellucida sperm-binding protein 2 (*ZP2*); Transcription factor IIIB (*TF3B*).
Sperm-egg recognition (GO:0035036, BP)	18 (35)	2.7E-06	Protein Bouncer (*BNCR*); CD9 antigen (*CD9*)
cytochrome complex (GO:0070069, CC)	29 (63)	7.5E-05	Cytochrome c oxidase subunit 3 (*COX3*); Cytochrome b (*CYB*); Normal mucosa of esophagus-specific gene 1 protein (*NMES1*); Cytochrome c oxidase subunit 1(*COX1*); Protoheme IX farnesyltransferase (*COX10*).
regulation of mitochondrial mRNA stability (GO:0044528, BP)	5 (5)	4.3E-03	FAST kinase domain-containing protein 3 (*FAKD3*); Phosphodiesterase 12 (*PDE12*).

regulation of reproductive process (GO:2000241, BP)	23 (93)	4.9E-03	F-box only protein 5 (*FBX5*); Histone-lysine N-methyltransferase (*PRDM9*); Putative transcription factor Ovo-like (*OVOL1*); Wee1-like protein kinase (*WEE2*); Cell division cycle protein 20 homolog (*CDC20*)

[1]Total number of DE genes is displayed in brackets. [2]FDR – False Discovery Rate (Benjamini and Hochberg, 1998)
